# Epigenetic profiling of Italian patients identified methylation sites associated with hereditary transthyretin amyloidosis

**DOI:** 10.1186/s13148-020-00967-6

**Published:** 2020-11-17

**Authors:** Antonella De Lillo, Gita A. Pathak, Flavio De Angelis, Marco Di Girolamo, Marco Luigetti, Mario Sabatelli, Federico Perfetto, Sabrina Frusconi, Dario Manfellotto, Maria Fuciarelli, Renato Polimanti

**Affiliations:** 1 Department of Biology, University of Rome Tor Vergata, Rome, Italy; 2 Department of Psychiatry, Yale University School of Medicine, VA CT Healthcare Center, VA CT 116A2, 950 Campbell Avenue, West Haven, CT USA; 3 VA CT Healthcare Center, West Haven, CT USA; 4 Clinical Pathophysiology Center, Fatebenefratelli Foundation –‘San Giovanni Calibita’ Fatebenefratelli Hospital, Rome, Italy; 5 Fondazione Policlinico Universitario A. Gemelli IRCCS, UOC Neurologia, Rome, Italy; 6 Università Cattolica del Sacro Cuore, Rome, Italy; 7 Centro Clinico NEMO Adulti, Rome, Italy; 8 Regional Amyloid Centre, Azienda Ospedaliero-Universitaria Careggi, Florence, Italy; 9 Genetic Diagnostics Unit, Laboratory Department, Careggi University Hospital, Florence, Italy

**Keywords:** hATTR, Amyloidosis, Val30Met mutation, Epigenetics, Methylation, Modifier gene

## Abstract

Hereditary transthyretin (TTR) amyloidosis (hATTR) is a rare life-threatening disorder caused by amyloidogenic coding mutations located in *TTR* gene. To understand the high phenotypic variability observed among carriers of *TTR* disease-causing mutations, we conducted an epigenome-wide association study (EWAS) assessing more than 700,000 methylation sites and testing epigenetic difference of *TTR* coding mutation carriers *vs*. non-carriers. We observed a significant methylation change at cg09097335 site located in *Beta-secretase 2* (*BACE2*) gene (standardized regression coefficient = −0.60, *p* = 6.26 × 10^–8^). This gene is involved in a protein interaction network enriched for biological processes and molecular pathways related to amyloid-beta metabolism (Gene Ontology: 0050435, *q* = 0.007), amyloid fiber formation (Reactome HSA-977225, *q* = 0.008), and Alzheimer’s disease (KEGG hsa05010, *q* = 2.2 × 10^–4^). Additionally, *TTR* and *BACE2* share APP (amyloid-beta precursor protein) as a validated protein interactor. Within *TTR* gene region, we observed that Val30Met disrupts a methylation site, cg13139646, causing a drastic hypomethylation in carriers of this amyloidogenic mutation (standardized regression coefficient = −2.18, *p* = 3.34 × 10^–11^). Cg13139646 showed co-methylation with cg19203115 (Pearson’s *r*^2^ = 0.32), which showed significant epigenetic differences between symptomatic and asymptomatic carriers of amyloidogenic mutations (standardized regression coefficient = −0.56, *p* = 8.6 × 10^–4^). In conclusion, we provide novel insights related to the molecular mechanisms involved in the complex heterogeneity of hATTR, highlighting the role of epigenetic regulation in this rare disorder.

## Background

Hereditary transthyretin amyloidosis (hATTR; OMIM#105210) is a life-threatening disorder caused by transthyretin (TTR) protein misfolding. This causes amyloid fibril deposition in several tissues (e.g., peripheral nerves, heart, and gastrointestinal tract) [1, 2]. hATTR is characterized by extreme clinical heterogeneity including age of onset, penetrance, and clinical display [3–5]. To date, more than 130 amyloidogenic mutations have been identified in the coding regions of the *TTR* gene, which are the cause of hATTR [6]. The prevalence of hATTR is estimated to be approximately 1/100,000 [7]. hATTR endemic areas have been identified in Portugal and Sweden [4, 5]. Although both of these regions are affected by the same amyloidogenic mutation, Val30Met (rs28933979), the penetrance and age of onset are different: early age of onset and high penetrance in Portugal [4, 5, 8, 9] *vs*. late age of onset and low penetrance in Sweden and in non-endemic countries [3, 10, 11]. hATTR phenotypic heterogeneity is likely due to the contribution of genetic and non-genetic factors involved in the complex genotype–phenotype correlation observed [12–18]. Recent data strongly support the role of non-coding regulatory variation on *TTR* gene expression, as one of the mechanisms affecting the phenotypic manifestations observed in carriers of *TTR* amyloidogenic mutations [19–22]. Among genomic regulatory features, epigenetic modifications are key mechanisms in modulating a wide range of molecular functions and potential targets to develop novel treatments [23–25]. Of several epigenetic modifications, DNA methylation is the most studied with respect to human traits and diseases [23]. In the context of monogenic disorders, methylation studies investigate the role of epigenetic changes involved in the phenotypic expression observed among carriers of disease-causing mutations [26–28]. While epigenetic modifications have the potential to be involved in hATTR pathogenic mechanisms, to our knowledge no study has explored methylation changes of patients affected by this life-threatening disease. In the present study, we conducted an epigenome-wide association study (EWAS) to identify DNA methylation sites associated with hATTR, investigating 48 carriers of *TTR* amyloidogenic mutations and 32 controls, comparing hATTR affected patients, asymptomatic carriers, and non-carriers.

### Results

**Fig. 1:**
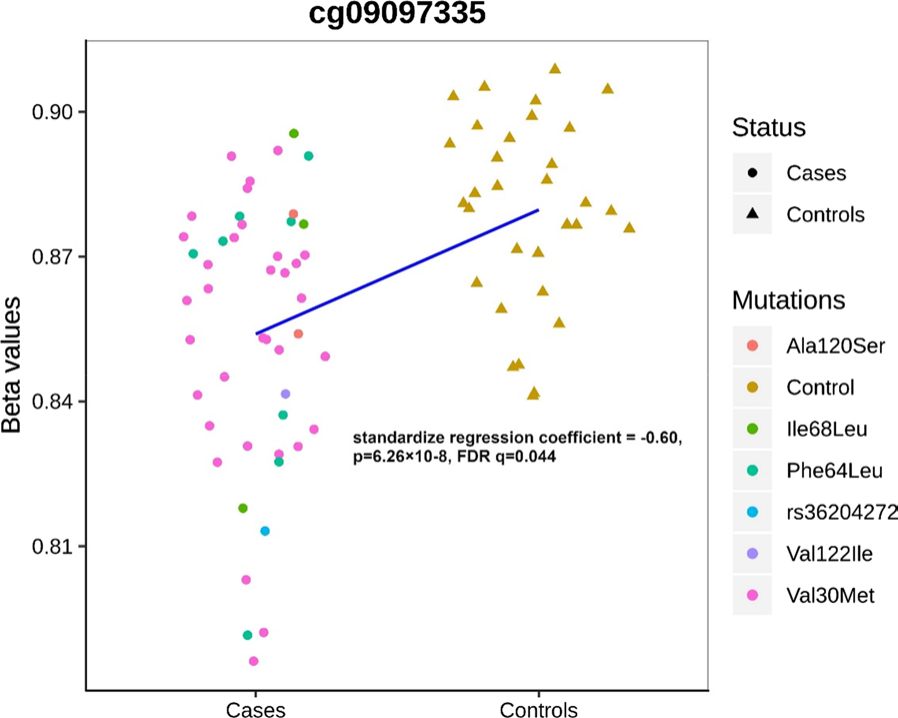
Methylation levels (beta-values) of cg09097335 site in carriers (cases) vs. non-carriers (controls) of amyloidogenic mutations. Regression line is shown in blue. The *M* values of this comparison are reported in Additional file 1. Standardized regression coefficient and *p* value reported are derived from the analysis conducted on the M values

**Fig. 2:**
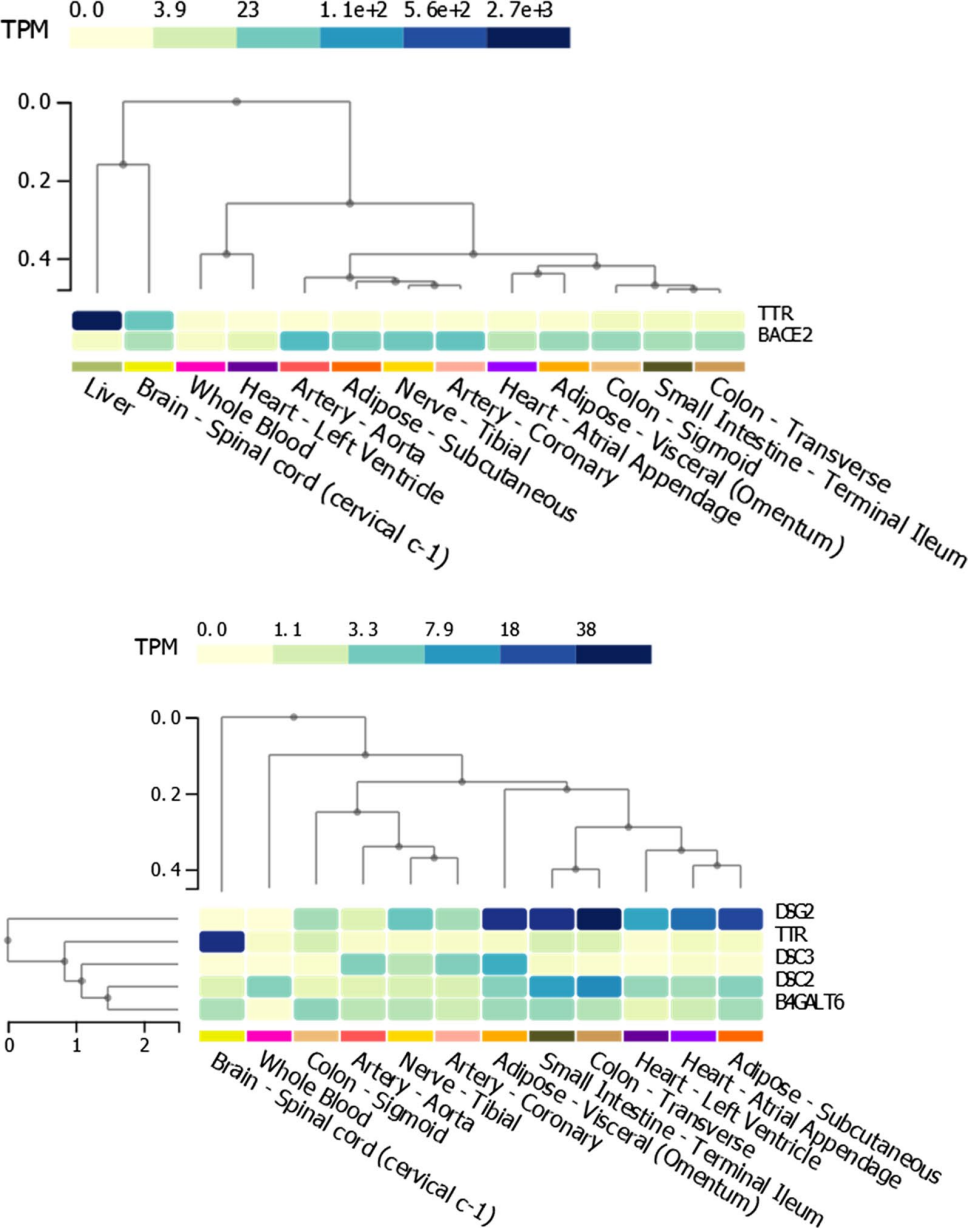
Upper Panel: Co-expression of *TTR* and *BACE2* in liver and in hATTR target organs; Bottom Panel: Co-expression of TTR, DSG2, DSC2, DSC3, and B4GALT6 in hATTR target organs (transcriptomic data from GTEx project, available at https://www.gtexportal.org/)

**Fig. 3:**
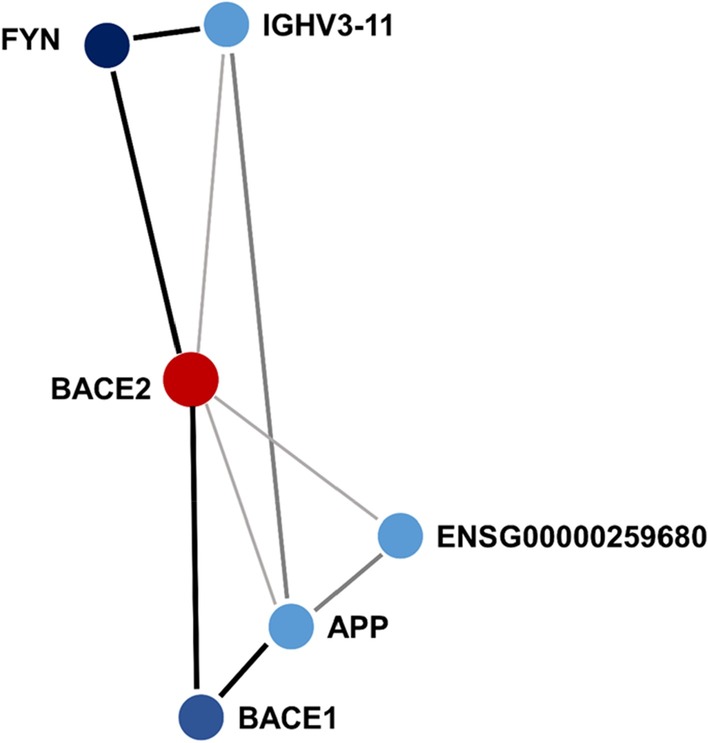
BACE2 protein interaction network. Node color of the protein is proportional to the interaction score with BACE2. Connector shade and width are proportional to the interaction confidence, highest, high, and medium

We compared the methylation changes (measured as M values, i.e., the log2 ratio of the intensities of methylated probe versus unmethylated probe; beta-values are also graphically presented to provide more biologically interpretable data) in the peripheral blood of 48 carriers of *TTR* amyloidogenic mutations and 32 controls (non-carriers). Testing more than 700,000 methylation sites, an association survived epigenome-wide false discovery rate correction (FDR *q* < 0.05) at the cg09097335 site located in *Beta-secretase 2* (*BACE2*) gene body (standardized regression coefficient = -0.60, *p* = 6.26 × 10^–8^, FDR *q* = 0.044). Carriers of *TTR* amyloidogenic mutations showed a significant hypomethylation when compared to controls (beta-value plot: Fig. 1; *M* value plot: Additional file 1). To understand whether methylation at this CpG site is associated with disease-associated genetic differences or post-disease processes, we compared hATTR patients, asymptomatic carriers of *TTR* mutations, and controls. Significant differences were observed for (i) hATTR patients *vs.* controls (standardized regression coefficient = −0.402, *p* = 5.7 × 10^–4^; Additional file 2, beta-value and *M* value plots) and ii) asymptomatic carriers vs. controls (standardized regression coefficient = −0.716, *p* = 3.21 × 10^–5^; Additional file 2: beta-value and *M* value plots), but no difference was present between hATTR patients *vs.* asymptomatic carriers (standardized regression coefficient = 0.137, *p* = 0.332). Similarly, considering different *TTR* mutations, we observed significant differences in cg09097335 methylation (i) between Val30Met carriers *vs.* controls (standardized regression coefficient = −0.587, *p* = 1.85 × 10^–6^; Additional file 2: beta-value and *M* value plots) and (ii) carriers of other *TTR* mutations *vs.* controls (standardized regression coefficient = −0.479, *p* = 1.27 × 10^–3^; Additional file 2: beta-value and *M* value plots), but not between Val30Met carriers *vs*. other *TTR* mutation carriers (standardized regression coefficient = 0.093, *p* = 0.424). Leveraging GTEx data [29], we observed a complementary transcriptomic regulation between *TTR* and *BACE2* genes where the first is mainly expressed in its source organ (i.e., liver), while the second is expressed in target organs of TTR amyloid deposits (Fig. 2, Upper Panel). We investigated interactive proteins related to *TTR* and *BACE2* loci based on multiple experimental and computational evidence, identifying five candidates with medium-to-highest interaction confidence (Fig. 3). These include FYN (FYN proto-oncogene, Src family tyrosine kinase; interaction score = 0.809), BACE1 (Beta-secretase 1; interaction score = 0.804), APP (amyloid-beta precursor protein; interaction score = 0.430), IGHV3-11 (immunoglobulin heavy variable 3–11; interaction score = 0.412), and ENSG00000259680 (uncharacterized protein similar to an immunoglobulin heavy variable 3/OR16 gene; interaction score = 0.412). Among them, TTR showed the highest interaction with APP protein (interaction score = 0.936). BACE2 protein interactive network (Fig. 3) showed functional enrichments for several biological processes and molecular pathways (Table 1). Among BACE2-related enrichments surviving FDR multiple testing correction, we observed: Alzheimer’s disease (AD, KEGG hsa05010, FDR *q* = 2.2 × 10^–4^) related to the interaction of BACE2 with APP and BACE1; membrane protein ectodomain proteolysis (GO: 0006509, FDR *q* = 0.007) and amyloid-beta metabolic process (GO: 0050435, FDR *q* = 0.007) related to BACE2-BACE1 interaction; protein metabolic process (GO: 0019538, FDR *q* = 0.043) related to the interaction of BACE with APP, BACE1, FYN, and IGHV3-11. The interactions of other proteins within BACE2 interactive network also highlighted amyloid-related functional enrichments: amyloid fiber formation (Reactome HSA-977225, FDR *q* = 0.008) and response to amyloid-beta (GO: 1904645, FDR *q* = 0.009) related to the interaction of APP with BACE1 and FYN, respectively.

**Table 1:** Enrichments for gene ontologies (GO) of biological processes and for Reactome and KEGG molecular pathways (HSA and hsa, respectively)

ID	Description	Proteins	False discovery rate q value
hsa05010	Alzheimer's disease	APP,BACE1,BACE2	2.2E-04
HSA-2029481	FCGR activation	FYN,IGHV3-11	7.9E-04
HSA-2730905	Role of LAT2/NTAL/LAB on calcium mobilization	FYN,IGHV3-11	7.9E-04
HSA-983695	Antigen activates B Cell Receptor (BCR) leading to generation of second messengers	FYN,IGHV3-11	0.002
GO: 0006509	Membrane protein ectodomain proteolysis	BACE1,BACE2	0.007
GO: 0050435	Amyloid-beta metabolic process	BACE1,BACE2	0.007
GO: 1902950	Regulation of dendritic spine maintenance	APP,FYN	0.007
HSA-977225	Amyloid fiber formation	APP,BACE1	0.008
GO: 1904645	Response to amyloid-beta	APP,FYN	0.009
HSA-109582	Hemostasis	APP,FYN,IGHV3-11	0.010
GO: 0106027	Neuron projection organization	APP,FYN	0.010
GO: 1900449	Regulation of glutamate receptor signaling pathway	APP,FYN	0.010
HSA-202733	Cell surface interactions at the vascular wall	FYN,IGHV3-11	0.010
GO: 0061098	Positive regulation of protein tyrosine kinase activity	APP,FYN	0.017
GO: 1903201	Regulation of oxidative stress-induced cell death	APP,FYN	0.017
GO: 0006897	Endocytosis	APP,FYN,IGHV3-11	0.018
GO: 0007631	Feeding behavior	APP,FYN	0.018
GO: 0016358	Dendrite development	APP,FYN	0.018
GO: 0038096	Fc-gamma receptor signaling pathway involved in phagocytosis	FYN,IGHV3-11	0.018
GO: 1901216	Positive regulation of neuron death	APP,FYN	0.018
GO: 1903426	Regulation of reactive oxygen species biosynthetic process	APP,FYN	0.018
GO:1900180	Regulation of protein localization to nucleus	APP,FYN	0.020
GO: 0007612	Learning	APP,FYN	0.027
GO: 0031347	Regulation of defense response	APP,FYN,IGHV3-11	0.027
GO: 2001056	Positive regulation of cysteine-type endopeptidase activity	APP,FYN	0.027
GO:0030162	Regulation of proteolysis	APP,FYN,IGHV3-11	0.031
GO: 0051897	Positive regulation of protein kinase B signaling	APP,FYN	0.031
GO: 2000377	Regulation of reactive oxygen species metabolic process	APP,FYN	0.031
HSA-168249	Innate Immune System	APP,FYN,IGHV3-11	0.032
HSA-76002	Platelet activation, signaling and aggregation	APP,FYN	0.032
GO: 0050808	Synapse organization	APP,FYN	0.034
GO: 1901215	Negative regulation of neuron death	APP,FYN	0.034
GO: 0002684	Positive regulation of immune system process	APP,FYN,IGHV3-11	0.037
GO: 0050776	Regulation of immune response	APP,FYN,IGHV3-11	0.037
GO: 0002252	Immune effector process	APP,FYN,IGHV3-11	0.041
GO: 0007411	Axon guidance	APP,FYN	0.041
GO: 0019538	Protein metabolic process	APP,BACE1,BACE2,FYN,IGHV3-11	0.043
GO: 0006959	Humoral immune response	APP,IGHV3-11	0.045

Within *TTR* gene region, we observed that Val30Met mutation disrupts a methylation site, cg13139646, causing a drastic hypomethylation in Val30Met carriers when compared with carriers of other *TTR* mutations (standardized regression coefficient = −2.18, *p* = 3.34 × 10^–11^; Additional file 3: beta-value and *M* value plots). Since Val30Met mutation disrupts the probe extension for the cg13139646 methylation site (the Met allele removes the G nucleotide from the CpG site), its impact on the methylation changes in *TTR* gene should be confirmed using alternative typing methods. However, to conduct an initial exploratory analysis of the potential functional implications of cg13139646 disruption, we performed a co-methylation analysis with respect to cg13139646 in the non-carrier sample. We identified 34 methylation sites that are correlated with cg13139646 site (Pearson’s *r*^2^ > 0.20; Additional file 4). Considering these co-methylated CpG sites, we investigated epigenetic differences among hATTR patients, asymptomatic carriers of *TTR* mutations, and controls (Additional file 5). Applying a Bonferroni correction accounting for the number of CpG sites tested, we observed a significant methylation difference between hATTR patients and asymptomatic carriers of *TTR* amyloidogenic mutations at cg19203115 (standardized regression coefficient = −0.555, *p* = 8.6 × 10^–4^; Additional file 6: beta-value and *M* value plots). Nominally significant methylation differences were observed (i) between carriers vs. non-carriers at cg11481443 (standardized regression coefficient = −0.306, *p* = 3.4 × 10^–3^; Additional file 6: beta-value and *M* value plots) and cg02936398 (standardized regression coefficient = 0.177, *p* = 4.9 × 10^–2^; Additional file 6: beta-value and *M* value plots); (ii) hATTR patients and asymptomatic carriers at cg14311811 (standardized regression coefficient = −0.273, *p* = 3.8 × 10^–2^; Additional file 6: beta-value and *M* value plots). Considering symptoms reported by hATTR patients, we identified CpG sites co-methylated with cg13139646 (i.e., the site disrupted by Val30Met mutation) nominally associated with cardiac involvement (cg27392998, standardized regression coefficient = −0.235, *p* = 7.5 × 10^–3^; cg18038361, standardized regression coefficient = 0.227, *p* = 5 × 10^–2^; Additional file 6: beta-value and *M* value plots), carpal tunnel syndrome (cg16492377, standardized regression coefficient = −0.229, *p* = 1.8 × 10^–2^; Additional file 6: beta-value and *M* value plots), and peripheral nervous system involvement (cg14719951, standardized regression coefficient = 0.249, *p* = 3.5 × 10^–2^; Additional file 6: beta-value and *M* value plots). Since some of these CpG sites were mapped to loci located near *TTR* gene (Additional file 5), we analyzed *TTR* transcriptomic profile in hATTR target organs, observing a different pattern when compared to the expression of the surrounding genes (Fig. 2, Bottom Panel).

### Discussion

hATTR is a rare multi-organ disorder caused by TTR misfolding and consequently amyloid deposition in several tissues [30]. This life-threatening condition is characterized by high clinical heterogeneity with respect to age of onset, penetrance, and phenotypic manifestation [1–10, 30]. Although *TTR* amyloidogenic mutations are the cause of *TTR* misfolding, non-coding variation and modifier genes are hypothesized to be involved in the wide variability of phenotypic manifestations observed in carriers of *TTR* disease-causing mutations [12, 15, 17–22]. Epigenetic modifications (e.g., DNA methylation changes) could also play an important role in the molecular network regulating the hATTR amyloidogenic process [25]. To explore this hypothesis, we conducted an EWAS investigating more than 700,000 methylation sites in 48 carriers of *TTR* amyloidogenic mutations and 32 non-carriers. A CpG site (cg09097335) located in *BACE2* gene was significantly hypomethylated in carriers when compared to non-carriers. This gene encodes Beta-secretase 2, a protein mainly known for its role in cleaving APP protein in amyloid-beta, which is a key factor involved in AD pathogenesis [31–33]. Differently from BACE1, which is the primary *β-secretase* protein cleaving APP to amyloid-beta, BACE2 is poorly expressed in the brain and its cleaving ability increases following an inflammatory response [34]. APP processing occurs via three proteolytic cleavages caused by α- β- and γ-secretase [35]. In non-amyloidogenic processes, α- and γ-secretases lead to the production of a smaller P3 fragment and APP intracellular domain, while, in the amyloidogenic pathway, β-secretase and γ-secretase produce amyloid-beta [35–39]. Our results also showed a high-confidence interaction between APP and TTR. Numerous studies explored the interactions between these two amyloidogenic proteins, displaying a relevant biological role of TTR in amyloid-beta aggregation and clearance in AD patients [40–44]. Specifically, TTR instability reduces the clearance of amyloid-beta, increasing amyloid toxicity in the brain [40–42]. Metal ions and interaction with other proteins could also affect TTR stability [40]. Although *TTR* genetic reduction did not alter APP processing, immunohistochemical and biochemical studies showed that genetic reduction of TTR elevates Aβ deposition in the brains of transgenic mice harboring APPswe/PS1ΔE9TTR + / − transgenes [45]. Under physiological conditions, the APP intracellular domain appears to be involved in epigenetically up-regulation of TTR to increase its amyloid-beta clearance activity [46]. In AD patients with *TTR* Val30Met, a significant association between amyloid-beta levels and AD was identified [40, 44]. A putative amyloidogenic role of amyloid-beta in hATTR was also identified in a post-mortem analysis of a Val30Met carrier where both TTR and amyloid-beta were deposited in the cerebral leptomeningeal and cortical blood vessel walls with a part of the vessel wall occupied by a combination of TTR and amyloid-beta aberrant proteins [43]. These previous findings strongly indicate an interplay between the pathogenic mechanisms involved in hATTR and AD. Our epigenome-wide study identified *BACE2* as a potential key factor in this interaction. As previously discussed, BACE2 protein plays a minor role in APP cleaving in the brain [33, 34], while its activity increases in peripheral tissues under inflammatory response [34]. Our transcriptomic analysis showed that *BACE2* is expressed in tissues affected by TTR amyloid deposits (i.e., heart, nerves, colon, small intestine, and adipose tissues). Accordingly, the methylation change observed in the TTR-mutation carriers is possibly due to the role of BACE2 in response to the inflammation induced by TTR amyloidogenic process in peripheral tissues [47].

Within *TTR* gene region, we observed that Val30Met disrupts a CpG site, causing a drastic hypomethylation in the carriers of this mutation. SNPs at CpG sites disrupting the methylation reactions can be associated with changes in regulatory function.[48, 49]. Although the impact of Val30Met on cg13139646 has to be confirmed by alternative methods because of the cg13139646 prove extension disruption, we conducted an initial exploratory analysis, testing the co-methylation of this site with CpG sites in the surrounding regions (NC_000018.9: 28,171,000–30,171,500). Indeed, co-methylation patterns reflect specific molecular mechanisms responsible for the regulation of multiple genes located in the same region [50]. In our analysis, some of the CpG sites identified map to *TTR* gene region, while others map in nearby loci. Considering hATTR target organs, these surrounding loci with co-methylated CpG sites have higher gene expression than *TTR*. We speculate that these genes may be involved in the formation of TTR amyloid deposits. This hypothesis is supported by the fact that co-methylated CpG sites are associated with hATTR traits. The strongest evidence was observed with respect to cg19203115 mapped in *B4GALT6* gene. Considering a Bonferroni correction accounting for the number of co-methylated CpG sites tested, cg19203115 showed a significant difference in methylation levels between hATTR patients and asymptomatic carriers of *TTR* mutations. *B4GALT6* gene encodes beta-1,4-galactosyltransferase 6, a type II membrane-bound glycoprotein that has exclusive specificity for the donor substrate UDP-galactose. B4GALT6 enzyme activity changes in response to inflammatory processes [51]. B4GALT6 stimulates astrocyte activation through the catalyzation of lactosylceramide synthesis, which in turn controls the production of pro-inflammatory cytokines and chemokines [51]. Hence, observing methylation changes in B4GALT6 may be associated with the inflammatory response to TTR amyloid deposits. Nominally significant differences were observed for CpG sites mapped in other surrounding loci: *DSC2* (cg02936398, Carriers *vs*. Controls); *DSG2* (cg14311811, hATTR patients *vs*. asymptomatic carriers); *DSC3* (cg16492377, carpal tunnel syndrome in hATTR patients). *DSC2* and *DSG2* encode components of the desmosome. This protein complex is specialized for cell-to-cell adhesion in myocardial tissue and mutations in *DSC2* and *DSG2* genes are associated with arrhythmogenic right ventricular cardiomyopathy [52]. In an in vivo study of myocardial inflammation, *DSC2* overexpression was observed to lead to tissue necrosis, fibrosis, and calcification of ventricles [53]. This process alters homeostasis among desmosomal proteins, inducing a cascade of different cell–cell interactions leading to cardiac remodeling [53]. In hATTR, cardiac amyloid fibril depositions also led to tissue dysfunctions [7]. Heart failure, restrictive cardiomyopathy, and rhythm disturbances (i.e., conduction system diseases, atrial fibrillation, and ventricular tachycardia) are the main clinical signs that occur after the accumulation of misfolded TTR protein [54–56]. Furthermore, transcriptomic interaction is observed between TTR and DSG2 to induce hypertrophic cardiomyopathy in animal models [57]. In this context, methylation changes in *DSC2* and *DSG2* genes could reflect pathogenic processes in hATTR target organs. We also identified two CpG sites co-methylated with cg13139646 (i.e., the methylation site disrupted by Val30Met mutation) that are nominally associated with hATTR symptoms. Cg18038361 is located in *TTR* gene promoter region and is associated with cardiac involvement in hATTR patients. DNA methylation changes in promoter regions are well known to play an important role in gene expression regulation [58, 59]. Cg18038361 association may be linked to regulatory changes in *TTR* gene expression. Lastly, two CpG sites—cg16492377 and cg14719951, map to *DSC3* transcription start site and gene body, respectively. In our analysis, methylation changes in these sites were associated with carpal tunnel syndrome and peripheral nervous system involvement in hATTR patients, respectively. *DSC3* gene encodes the desmocollin-3 a calcium-dependent glycoprotein. Low *DSC3* expression in human epidermis leads to a loss of tissue integrity [60]. We speculate that cg16492377 methylation association with carpal tunnel syndrome may be related to changes in *DSC3* transcriptomic regulation.

Although we provide novel findings regarding the role of methylation changes in hATTR, our study presents several limitations. Since hATTR is a rare disease, we investigated a relatively small sample size. Our calculation showed that the sample size investigated in our main analysis should provide > 80% statistical power to detect medium effect sizes (Δ_*β*_ = 0.2; Additional file 7). However, large samples will be needed to investigate how epigenetic changes affect hATTR symptoms and differences across *TTR* amyloidogenic mutations. Our cohort showed age and sex differences between carriers of *TTR* amyloidogenic mutations and controls. Our analysis was adjusted for these confounding variables together with blood cell types, genetic principal components, and epigenetically determined smoking status. More balanced case–control groups are needed to investigate how epigenetic differences are associated differently between sexes and across age groups. We used transcriptomic data from GTEx project to explore the potential mechanisms related to the epigenetic associations identified. Further studies generating transcriptomic and epigenomic information across multiple informative tissues will provide a more comprehensive understanding of the molecular processes involved in hATTR. Although we provide some preliminary evidence showing that Val30Met mutation disrupts cg13139646 which appears co-methylated with CpG sites potentially associated with hATTR, our findings should be considered exploratory. Indeed, since Val30Met disrupts the probe extension for the cg13139646 methylation site, the impact of this mutation on the methylation changes in *TTR* gene should be confirmed using alternative methods.

## Conclusions

Our study provided novel insights regarding hATTR pathogenesis, supporting the involvement of methylation changes in the amyloidogenic process induced by *TTR* disease-causing mutations. Further studies will be needed to characterize specific mechanisms underlying the epigenetic associations with particular attention to the potential role of amyloid-beta metabolic process and inflammatory response. The understanding of how methylation changes modulate the penetrance and the severity of *TTR* mutations could lead to the identification of novel targets to develop treatments and screening tools for the carriers. Additionally, similarly to what was observed with respect to genetic variation [21], it will be important to estimate the epigenetic similarity between hATTR and wild-type transthyretin amyloidosis.

## Methods

Thirty-eight symptomatic patients and 10 asymptomatic *TTR* mutations carriers were recruited from three Italian centers for the treatment of systemic amyloidosis: “San Giovanni Calibita” Fatebenefratelli Hospital, Isola Tiberina—Rome, Fondazione Policlinico Universitario “A. Gemelli”—Rome, and Careggi University Hospital—Florence [16–20]. Thirty-two controls were recruited by the Department of Biology—University of Rome “Tor Vergata” (Table 2). hATTR diagnosis was based on the presence of clinical signs and symptoms and the presence of an amyloidogenic mutation on *TTR* gene. One hATTR patient is a carrier of a mutation (rs36204272) in an intronic region with a putative clinical impact [61]. Carpal tunnel syndrome and cardiac involvement with confirmed TTR amyloid deposits are present in rs36204272 carrier. Information regarding the organ involvements was collected for each patient: peripheral and nerve involvement (nerve conduction study); cardiac involvement (electrocardiographic and echocardiography anomalies); gastrointestinal involvement (gastric paresis, stypsis, or diarrhea); autonomic neurological involvement (orthostatic hypotension and urinary incontinence); ocular involvement (vitreous opacities): and carpal tunnel syndrome (median nerve decompression) [11, 62–64]. In our previous analysis in this cohort [20], we observed that certain mutations were associated with specific clinical manifestations: Ile68Leu and Val122Ile with cardiac involvement; Val30Met with ocular symptoms; Phe64Leu with renal involvement. Conversely, autonomic neurological, peripheral neurological, and gastrointestinal symptoms were observed in most of the amyloidogenic mutations [20]. The present study was approved under the protocol 39/18 by the Comitato Etico Indipendente, Fondazione Policlinico Tor Vergata—Rome, Italy.

**Table 2:** Description of the study population. Information about *TTR* amyloidogenic mutations, sex, age, epigenetically determined smoking status (never smoker, NS; former smoker, FS; current smoker, CS), and epigenetically estimated ranges of T cells (CD8T and CD4T), natural killer cells (NK), B cells, monocytes (Mono) and granulocytes (Gran) are reported

TTR mutation	*N*	Sex Female (%)	Age median (Min–Max)	Smoking			CD8T Median (Min –Max)	CD4T median (Min–Max)	NK median (Min–Max)	B cell median (Min–Max)	Mono median (Min–Max)	Gran median (Min–Max)
				NS	FS	CS						
*Cases*												
Val30Met	33	10 (30)	65 (31–88)	1	23	9	0.033 (−3.24 × 10^–20^–0.131)	0.118 (1.1 × 10^–2^–0.21)	0.033 (−3.81 × 10^–19^–0.127)	1.52 × 10^–3^ (−5.55 × 10^–19^–0.066)	0.082 (0.038–0.147)	0.634 (0.551–0.824)
Phe64Leu	8	1 (12)	70 (48–75)	0	4	4	4.02 × 10^–3^ (1.07 × 10^–18^–1.112)	0.127 (6.9 × 10^–2^–0.228)	0.014 (1.96 × 10^–20^–0.099)	3.13 × 10^–3^ (−2.17 × 10^–19^–0.021)	0.085 (0.057–0.107)	0.65 (0.46–0.704)
Ala120Ser	2	2 (100)	67–68	0	2	0	−3.34 × 10^–20^–0	0.068–0.11	8.1 × 10^–3^–1.28 × 10^–1^	0–5.1 × 10^–3^	0.092–0.11	0.56–0.74
Ile68Leu	3	0	53 (30–62)	0	0	3	0	0.142 (0.07–0.16)	0.038 (0.013–0.041)	4.4 × 10^–3^ (3.8 × 10^–3^–5.1 × 10^–3^)	0.083 (0.096–0.147)	0.59 (0.603–0.73)
Val122Ile	1	0	80	0	0	1	0	0.23	0.042	0.005	0.071	0.55
rs36204272*	1	0	65	0	1	0	0.069	0.14	0.052	0.006	0.129	0.505
*Controls*												
–	32	19 (59)	37 (20–76)	0	27	5	0.015 (8.45 × 10^–19^–0.13)	0.16 (0.045–0.34)	0.044 (−2.68 × 10^–19^–0.18)	0.014 (1.08 × 10^–19^–0.091)	0.092 (0.051–0.13)	0.571 (0.41–0.76)

^*^Non-coding variant of putative clinical impact

### DNA methylation analysis

DNA was extracted using the phenol/chloroform protocol [65] and purified through Amicon Ultra-0.5 mL Centrifugal Filters (EMD Millipore) to achieve a DNA concentration of 100 ng/µL. DNA concentration was checked via NanoDrop technology (ND-1000, Thermo Fisher Scientific) and Qubit Quantitation technology (High Accuracy & Sensitivity, Thermo Fisher). DNA methylation analysis was executed in two phases: the EZ DNA Methylation kit (Zymo Research) was used to perform sodium bisulfite conversion; the Illumina Infinium Methylation EPIC Chip (with over 850,000 methylation sites; Illumina Inc.) was used to quantify DNA methylation according to the standard Illumina protocol. The methylation array analysis was performed at the Connecting bio-research and Industry Center, Trieste—Italy.

### Preprocessing, quality control, and normalization

The raw signal intensity files were processed and cleaned using R 3.6 and ChAMP package [66]. The ratio of methylated and unmethylated intensities from idat files was converted into beta-values for further processing. The probes failing thresholds on detection *p* value (< 0.01) bead count, sites near SNPs—this method performed extensive characterization of probes on the EPIC and HM450 microarrays, including mappability to the latest genome build, genomic copy number of the 3΄ nested subsequence, and influence of polymorphisms including a previously unrecognized color channel switch for Type I probes, probes that align to multiple positions, sex chromosomes and outliers were removed. None of the samples failed quality control. The remaining 718,509 probes for 80 individuals were normalized with BMIQ. Batch effects were assessed using singular vector decomposition and corrected with ComBat method [67]. The genomic lambda of the case–control association was 1.03, calculated using QQPerm package (https://cran.r-project.org/web/packages/QQperm/index.html).

### Blood cell type composition, genetic variability estimation, and smoking prediction

A reference-based method was employed to adjust for the heterogeneity due to the cell type composition of the whole blood samples investigated [68]. This method uses specific DNA methylation signatures derived from purified whole blood cell type as biomarkers of cell identity, to correct the beta-values. Cell proportions for five cell types (B cells, granulocytes, monocytes, natural killer cells, and T cells) were detected, and a linear regression was applied [66, 68]. To account for the genetic variability among the samples investigated, principal components (PCs) were calculate using the method proposed by Barfield, Almli [69]. This approach allowed us to compute PCs based on CpGs selected for their proximity to SNPs. The data obtained can be used to adjust for population stratification in DNA methylation studies when genome-wide SNP data are unavailable [69]. Cigarette smoke has a very large effect on DNA methylation profile, triggering alteration at multiple CpGs [70]. Consequently, smoking status needs to be considered as a potential confounder in epigenetic association studies. EpiSmokEr package was used to classify the smoking status of each participant on the basis of their epigenetic profile [71]. Briefly, EpiSmokEr is a prediction tool that provides smoking probabilities for each individual (never smoker, former smoker, and current smoker) using a set of 121 informative CpG sites [70].

### Data analysis

We conducted an epigenome-wide analysis testing 718,509 methylation sites. First, we investigated the methylation changes (measured as M values, i.e., the log2 ratio of the intensities of methylated probe versus unmethylated probe; beta-values are plotted to provide more biologically interpretable data) between 48 carriers of a *TTR* amyloidogenic mutation) and 32 controls (i.e., non-carriers). Considering CpG sites that survive epigenome-wide multiple testing correction, we verified whether the associations observed were due to disease-associated genetic differences or post-disease processes, comparing (i) patients affected by hATTR *vs*. controls, (ii) asymptomatic carriers of *TTR* mutations *vs*. controls, and iii) patients affected by hATTR *vs*. asymptomatic carriers of *TTR* mutations. Additionally, we also tested whether the methylation changes observed in the case–control analysis were different between (i) Val30Met carriers vs. controls, (ii) carriers of other *TTR* mutations vs. controls and, iii) Val30Met patients vs. carriers of other *TTR* mutations.

To investigate the functionality of the CpG disrupted by Val30Met mutation, we analyzed its co-methylation with CpG sites in the surrounding region (NC_000018.9: 28,171,000–30,171,500). This region was selected based on the *TTR* regulatory mechanisms observed in previous studies [20–22]. We used *cor()* R function to calculate Pearson’s correlation coefficient testing 367 sites and considering the methylation levels (*M* values) using as reference the non-carriers. CpG sites with high co-methylation (Person’s *r*^2^ > 0.2) were investigated with respect to hATTR-related traits (i.e., carrier status, disease status, and symptoms). In all association analyses, we implemented a linear regression analysis including cell composition proportions, top three genetic PCs, epigenetically determined smoking status, age, and sex as covariates. The results of the regression analyses were reported as standardized regression coefficients and p values. FDR method [72] was applied to adjust the results for epigenome-wide testing and the *q* value < 0.05 was considered as the significance threshold. Co-expression analysis was conducted using GTEx v8 [29] via the Multi-Gene Query available at https://www.gtexportal.org/. *Ggplot2* R package [73] was employed to plot co-methylation pattern results. STRING v.11.0 [74] was used to identify protein interaction with the loci identified, considering experiments, co-expression, co-occurrence, gene fusion, and neighborhood as active sources and an interaction score higher than 0.4 (medium confidence). The protein interaction network was investigated further conducting functional enrichments association related to the protein–protein interactions identified considering Gene Ontologies [75] for biological processes and molecular pathways available from Reactome Database [76] and Kyoto Encyclopedia of Genes and Genomes (KEGG) [77]. FDR (*q* value < 0.05) was applied to account for multiple testing assuming the whole genome as the statistical background. A statistical power calculation was done using pwrEWAS tool [78] considering medium and small effect sizes (Δ_*β*_ = 0.5 and 0.2, respectively) and multiple sample sizes.

## Supplementary information

**Additional file 1**. Methylation levels (M values) of cg09097335 site in carriers (cases) vs. non-carriers (controls) of amyloidogenic mutations. The regression line is shown in blue.

**Additional file 2**. Methylation change of cg09097335 site (upper panel: beta values; lower panel: M values) between i) hATTR patients (carriers of TTR amyloidogenic mutations with hATTR diagnosis) vs. controls, ii) asymptomatic carriers vs. controls, iii) V30M carriers vs. controls, and iv) carriers of other TTR mutation vs. controls. Standardized regression coefficient and p value reported for each comparison are derived from the analysis conducted on the M values.

**Additional file 3**. Methylation change of cg13139646 site between V30M carriers vs. controls (upper panel: beta values; bottom panel: M values). Standardized regression coefficient and p value reported for each comparison are derived from the analysis conducted on the M values.

**Additional file 4**. Co-methylation analysis (Pearson’s correlation based on M values) with respect to cg13139646 (red text).

**Additional file 5**. Association of cg13139646 co-methylated CpG sites (Pearson’s r2 > 0.2) with i) carrier status (cases vs. controls), ii) disease status (cases vs. asymptomatic) and, iii) symptoms (AGE, ANS, CTS, EYE, GI, HEA, PNS). Information about cg probe (cgID), chromosome localization (CHR), position (POS), determination coefficient (Pearson’s r2), standardized regression coefficients (Reg. Coef.), p value (Pval), mapped gene (GENE), gene region (Genomic Region), are reported. AGE = age of onset; ANS = autonomic nervous system; CTS = carpal tunnel syndrome; EYE = ocular involvement; GI = gastrointestinal involvement; HEA = cardiac involvement; PNS = peripheral nervous system. Red and underlined text = significant results surviving Bonferroni correction. Red text = nominally significant results.

**Additional file 6**. Methylation changes of co-methylated cg13139646-correlated CpG sites with respect to hATTR-related phenotypes: cg19203115, hATTR patients vs. asymptomatic carriers; cg11481443, cases vs. controls; cg02936398, cases vs. controls; cg14311811, hATTR patients vs. asymptomatic carriers; cg27392998, cardiac involvement in hATTR patients; cg18038361, cardiac involvement in hATTR patients; cg16492377, carpal tunnel syndrome in hATTR patients; cg14719951, peripheral nervous system involvement in hATTR patients.

**Additional file 7**. Statistical power calculations based on medium and small effect sizes (Δ_β_ = 0.5 and 0.2, respectively) and multiple sample sizes.

## Data Availability

Data supporting the findings of this study are available within this article and its additional files.

## Abbreviations

AD: Alzheimer disease
APP: Amyloid-beta precursor protein
hATTR: Hereditary transthyretin amyloidosis
BACE1: Beta-secretase 1
B4GALT6: Beta-1,4-galactosyltransferase 6
BACE2: Beta-secretase 2
DSC2: Descmocollin-2
DSC3: Desmocollin-3
DSG3: Desmoglein-3
EWAS: Epigenome-wide association studies
FDR: False discovery rate
FYN: FYN proto-oncogene, Src family tyrosine kinase
IGHV3-11: Immunoglobulin heavy variable 3–11
TTR: Transthyretin
