# Supplementary material for: Epigenetic profiling of Italian patients identified methylation sites associated with hereditary transthyretin amyloidosis
Source: Clin Epigenetics. 2020 Nov 17;12:176. doi: 10.1186/s13148-020-00967-6 (PMC7672937; doi:10.1186/s13148-020-00967-6)

**Additional File 1**: Methylation levels (M values) of cg09097335 site in carriers (cases) vs. non-carriers (controls) of amyloidogenic mutations. The regression line is shown in blue.


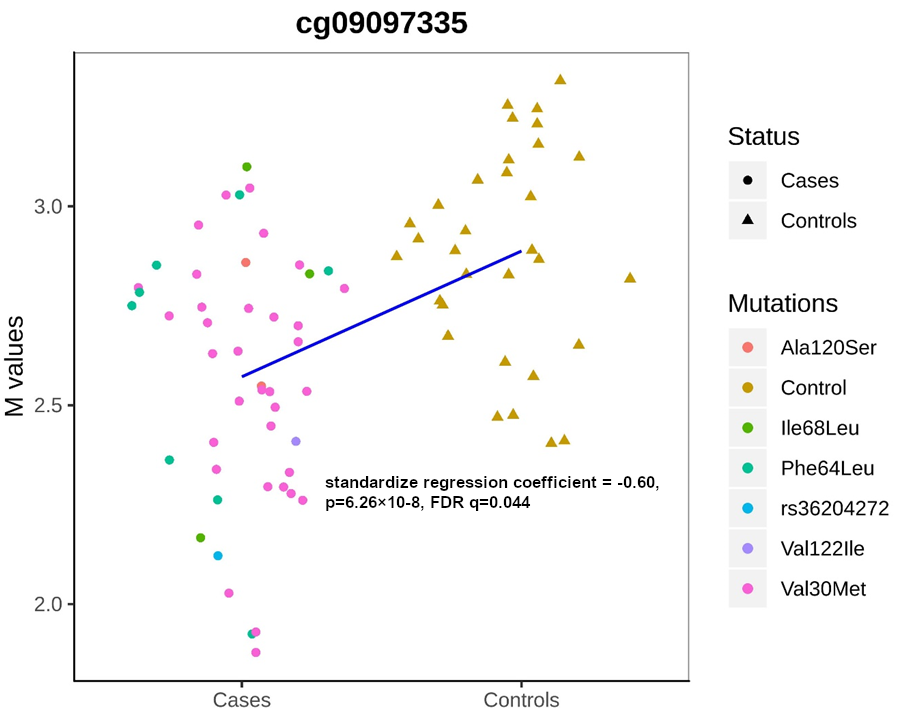

Supplement: Supplementary file 1 — Additional file 1. Methylation levels (M values) of cg09097335 site in carriers (cases) vs. non-carriers (controls) of amyloidogenic mutations. The regression line is shown in blue. [file 13148_2020_967_MOESM1_ESM.docx]
