# Supplementary material for: Epigenetic profiling of Italian patients identified methylation sites associated with hereditary transthyretin amyloidosis
Source: Clin Epigenetics. 2020 Nov 17;12:176. doi: 10.1186/s13148-020-00967-6 (PMC7672937; doi:10.1186/s13148-020-00967-6)

**Additional File 2:** Methylation change of cg09097335 site (upper panel: beta values; lower panel: M values) between i) hATTR patients (carriers of *TTR* amyloidogenic mutations with hATTR diagnosis) vs. controls, ii) asymptomatic carriers vs. controls, iii) V30M carriers vs. controls, and iv) carriers of other *TTR* mutation vs. controls. Standardized regression coefficient and p value reported for each comparison are derived from the analysis conducted on the M values.


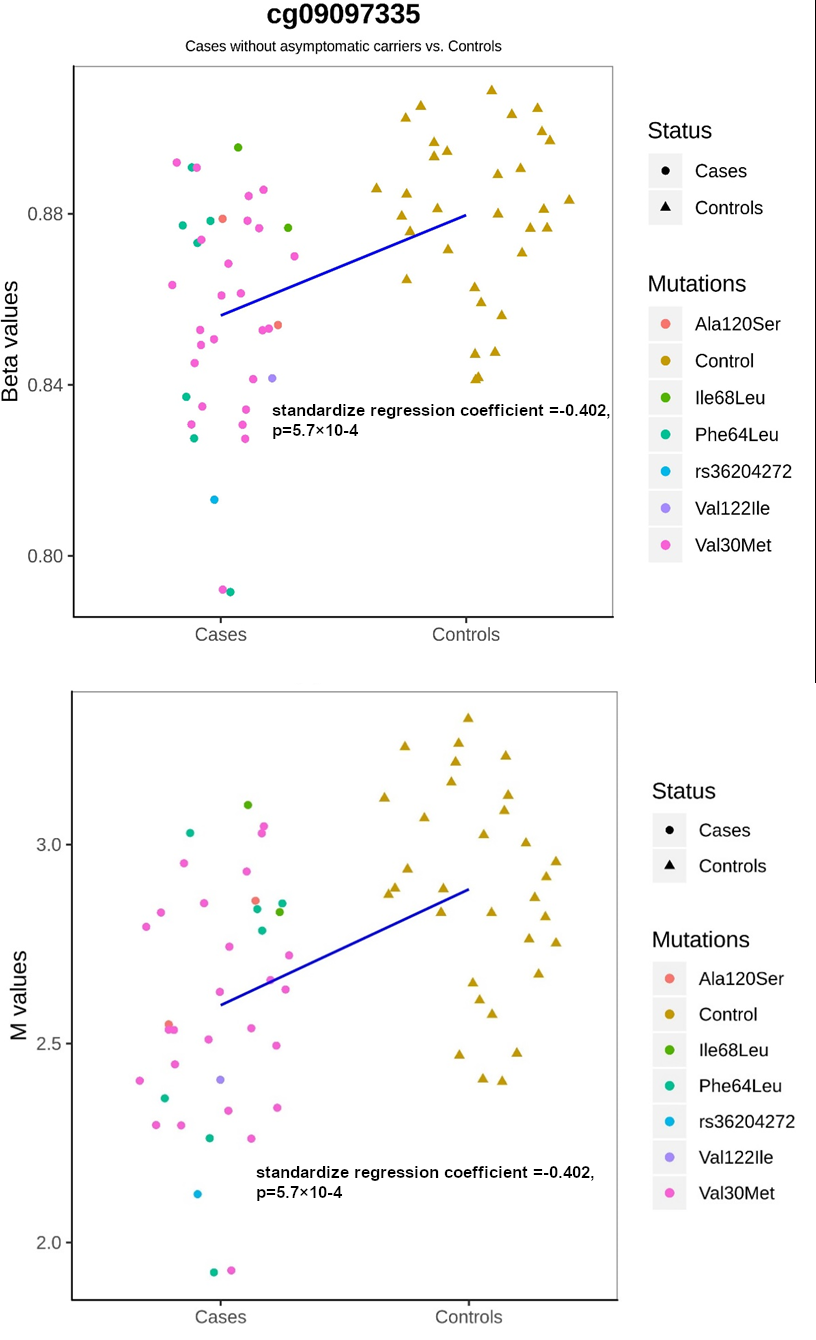


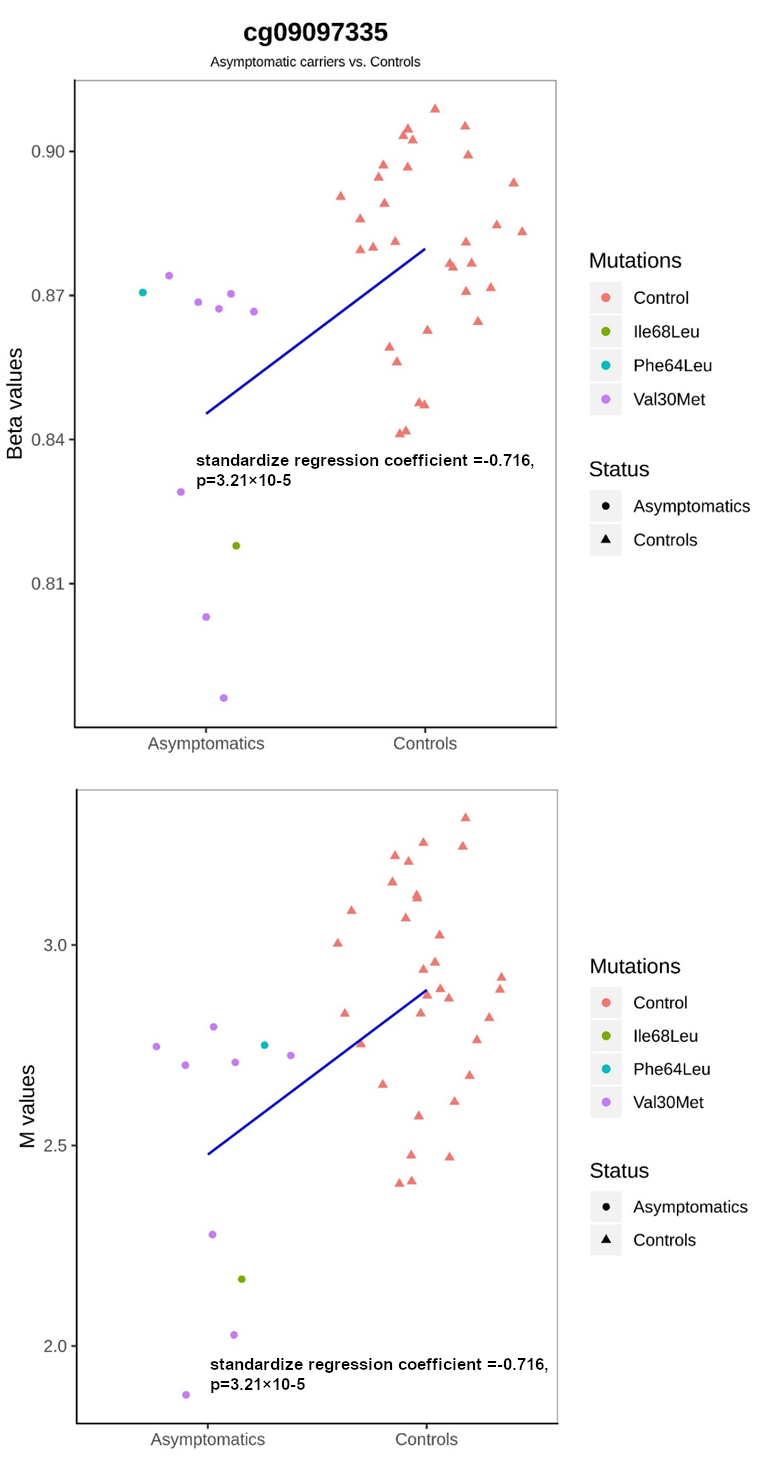


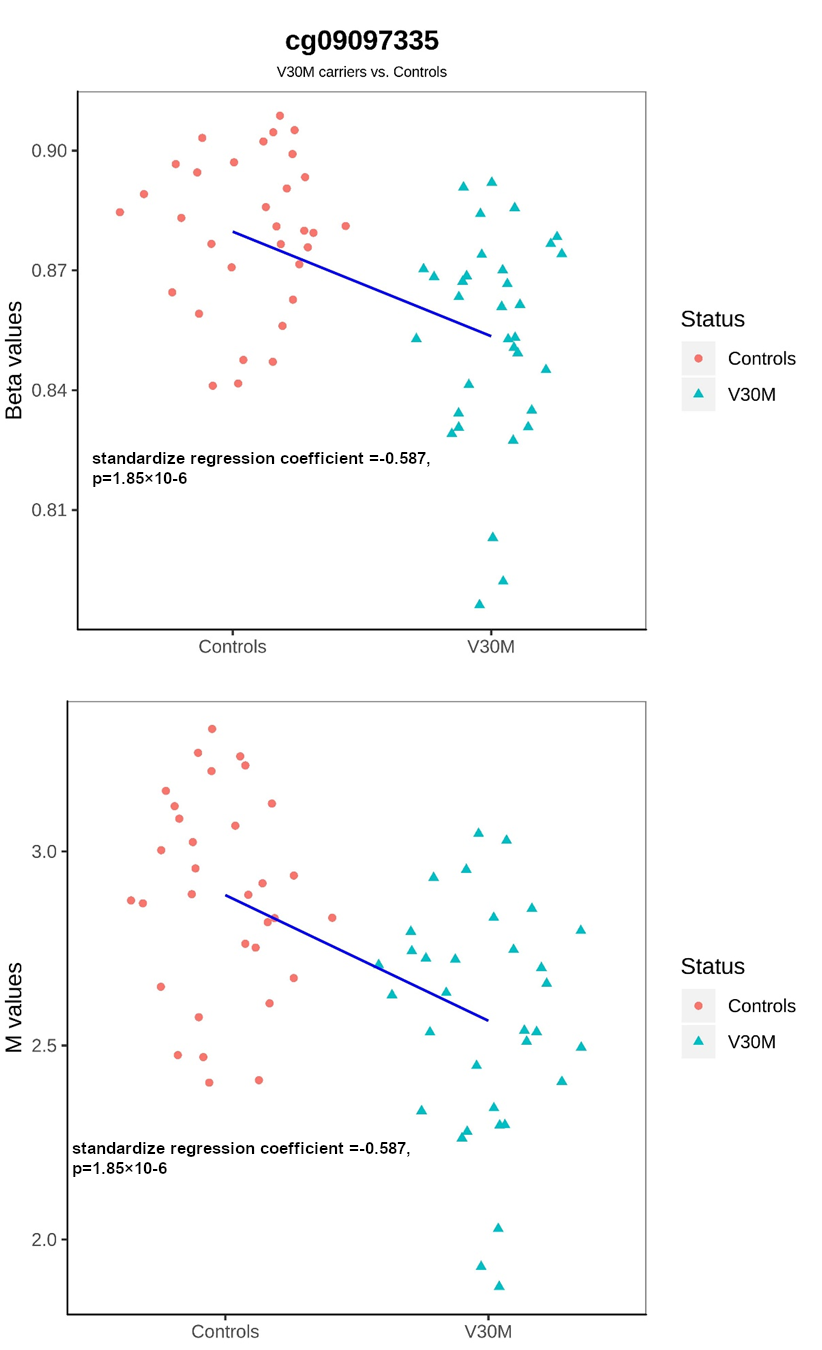


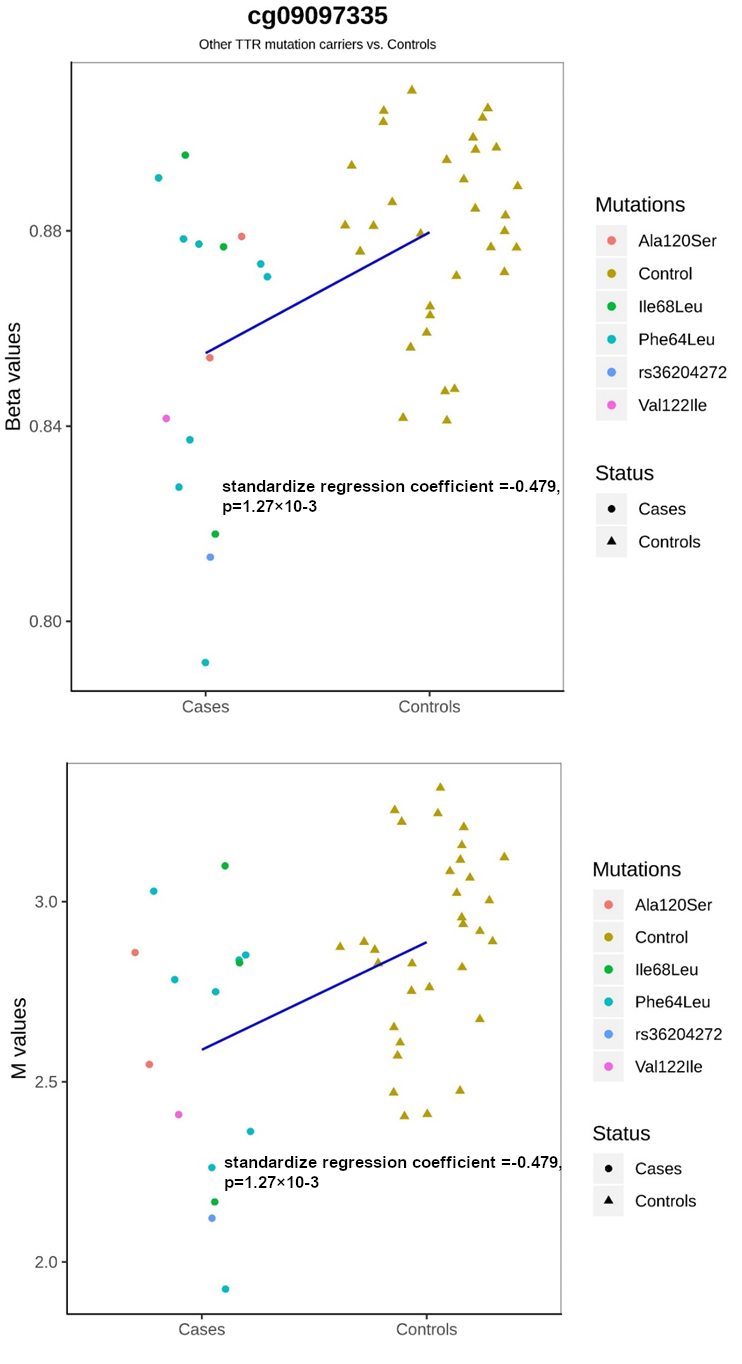

Supplement: Supplementary file 2 — Additional file 2. Methylation change of cg09097335 site (upper panel: beta values; lower panel: M values) between i) hATTR patients (carriers of TTR amyloidogenic mutations with hATTR diagnosis) vs. controls, ii) asymptomatic carriers vs. controls, iii) V30M carriers vs. controls, and iv) carriers of other TTR mutation vs. controls. Standardized regression coefficient and p value reported for each comparison are derived from the analysis conducted on the M values. [file 13148_2020_967_MOESM2_ESM.docx]
