# Supplementary material for: Epigenetic profiling of Italian patients identified methylation sites associated with hereditary transthyretin amyloidosis
Source: Clin Epigenetics. 2020 Nov 17;12:176. doi: 10.1186/s13148-020-00967-6 (PMC7672937; doi:10.1186/s13148-020-00967-6)

**Additional File 3:** Methylation change of cg13139646 site between V30M carriers vs. controls (upper panel: beta values; bottom panel: M values). Standardized regression coefficient and p value reported for each comparison are derived from the analysis conducted on the M values.


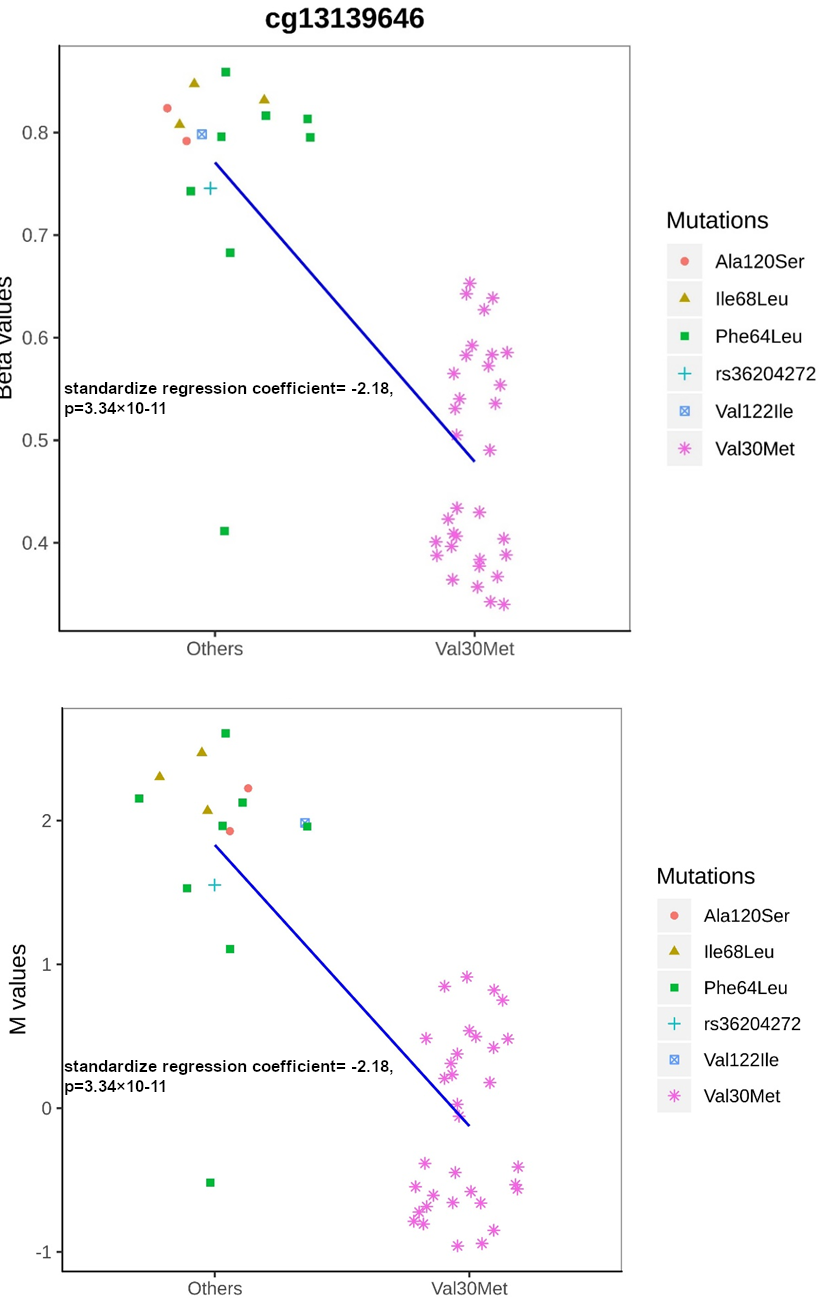

Supplement: Supplementary file 3 — Additional file 3. Methylation change of cg13139646 site between V30M carriers vs. controls (upper panel: beta values; bottom panel: M values). Standardized regression coefficient and p value reported for each comparison are derived from the analysis conducted on the M values. [file 13148_2020_967_MOESM3_ESM.docx]
