# Supplementary material for: Epigenetic profiling of Italian patients identified methylation sites associated with hereditary transthyretin amyloidosis
Source: Clin Epigenetics. 2020 Nov 17;12:176. doi: 10.1186/s13148-020-00967-6 (PMC7672937; doi:10.1186/s13148-020-00967-6)
Supplement: Supplementary file 4 — Additional file 4. Co-methylation analysis (Pearson’s correlation based on M values) with respect to cg13139646 (red text). [file 13148_2020_967_MOESM4_ESM.docx]

**
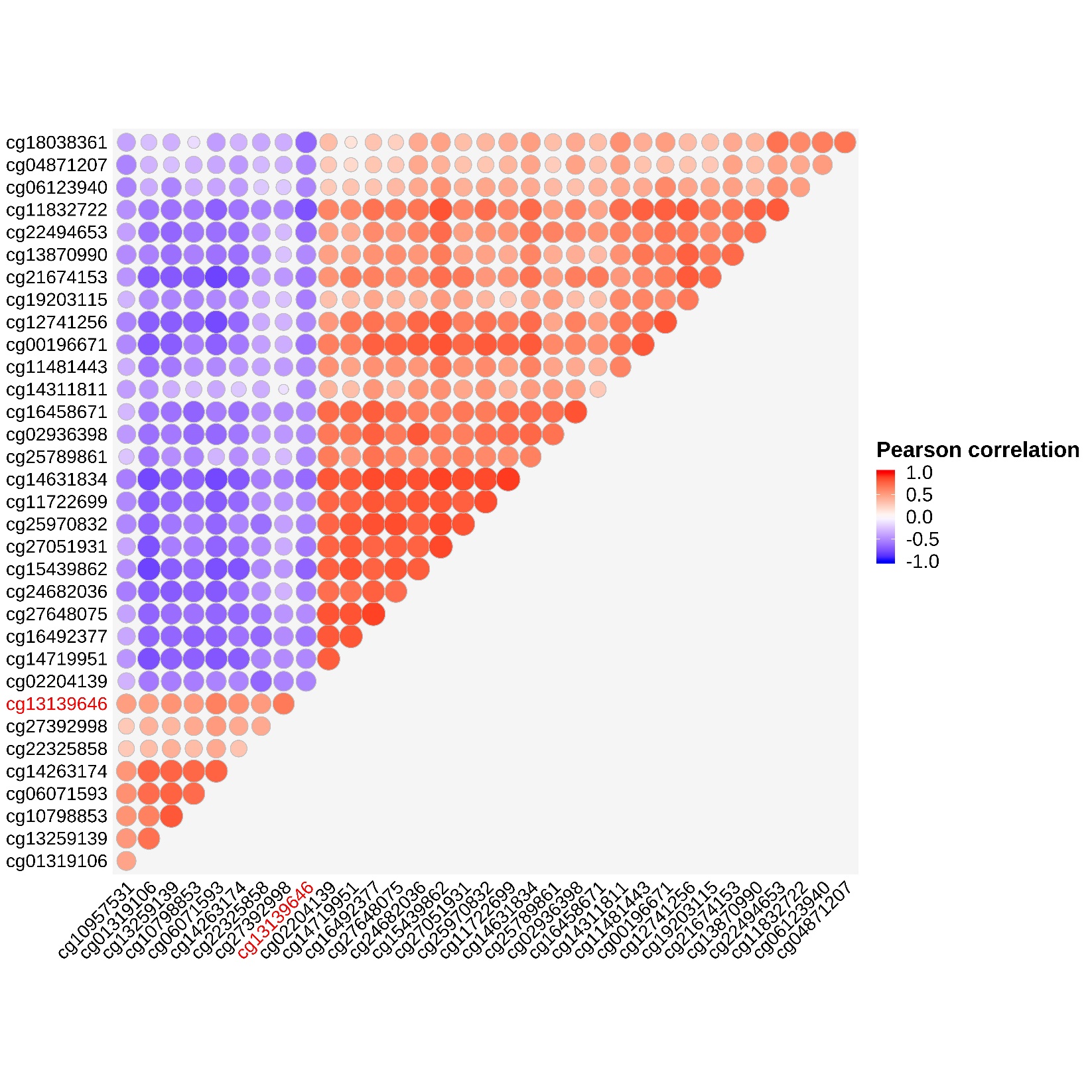
Additional File 4:** Co-methylation analysis (Pearson’s correlation based on M values) with respect to cg13139646 (red text).
