# Supplementary material for: Epigenetic profiling of Italian patients identified methylation sites associated with hereditary transthyretin amyloidosis
Source: Clin Epigenetics. 2020 Nov 17;12:176. doi: 10.1186/s13148-020-00967-6 (PMC7672937; doi:10.1186/s13148-020-00967-6)

**Additional File 6:** Methylation changes of co-methylated cg13139646-correlated CpG sites with respect to hATTR-related phenotypes: cg19203115, hATTR patients *vs.* asymptomatic carriers; cg11481443, cases vs. controls; cg02936398, cases vs. controls; cg14311811, hATTR patients vs. asymptomatic carriers; cg27392998, cardiac involvement in hATTR patients; cg18038361, cardiac involvement in hATTR patients; cg16492377, carpal tunnel syndrome in hATTR patients; cg14719951, peripheral nervous system involvement in hATTR patients.


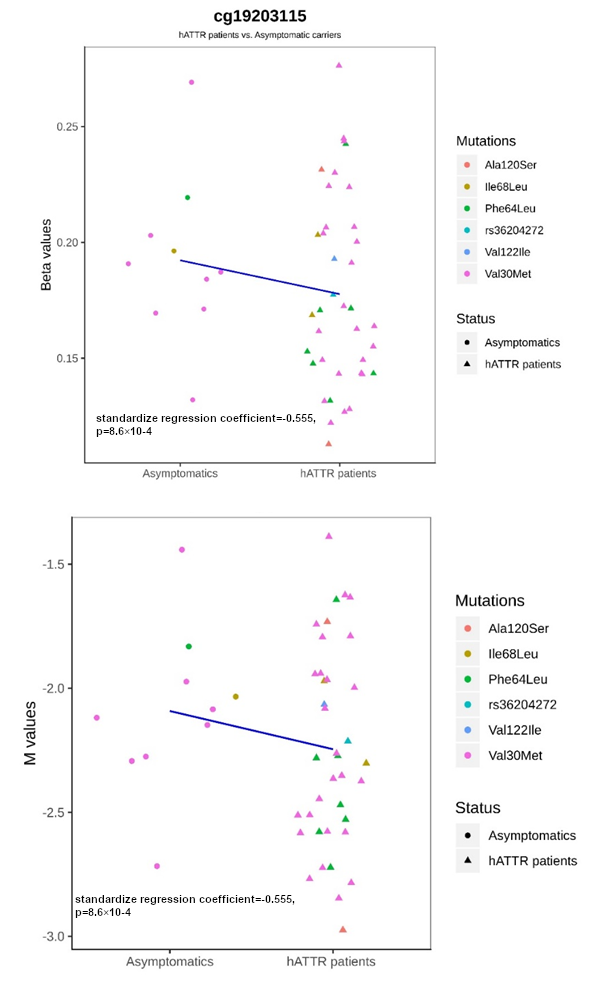


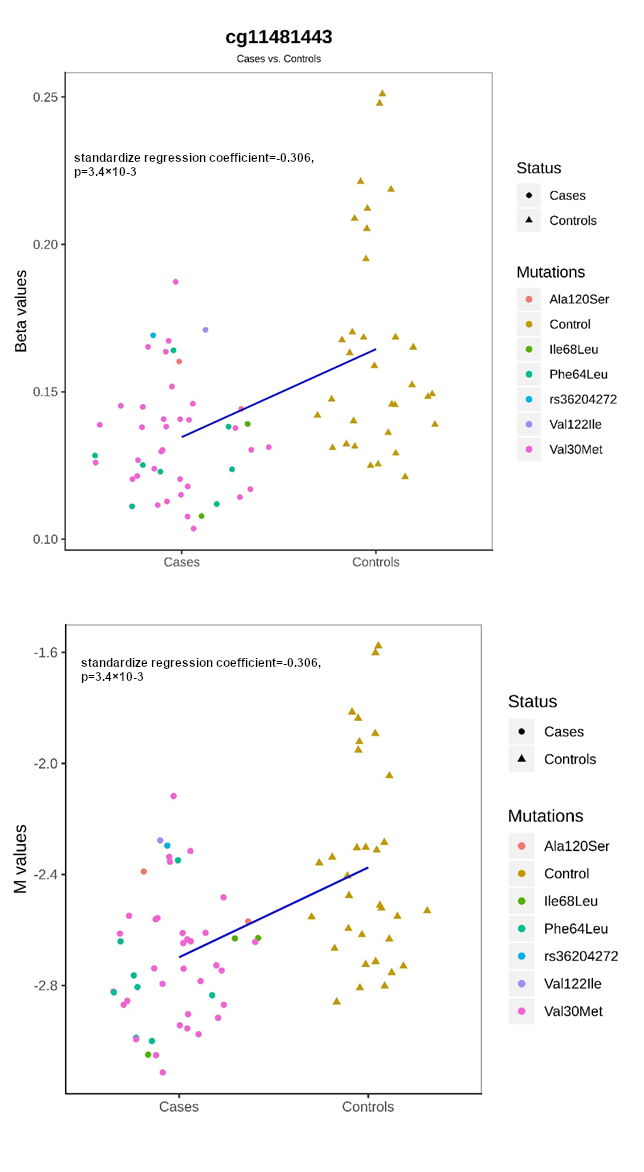


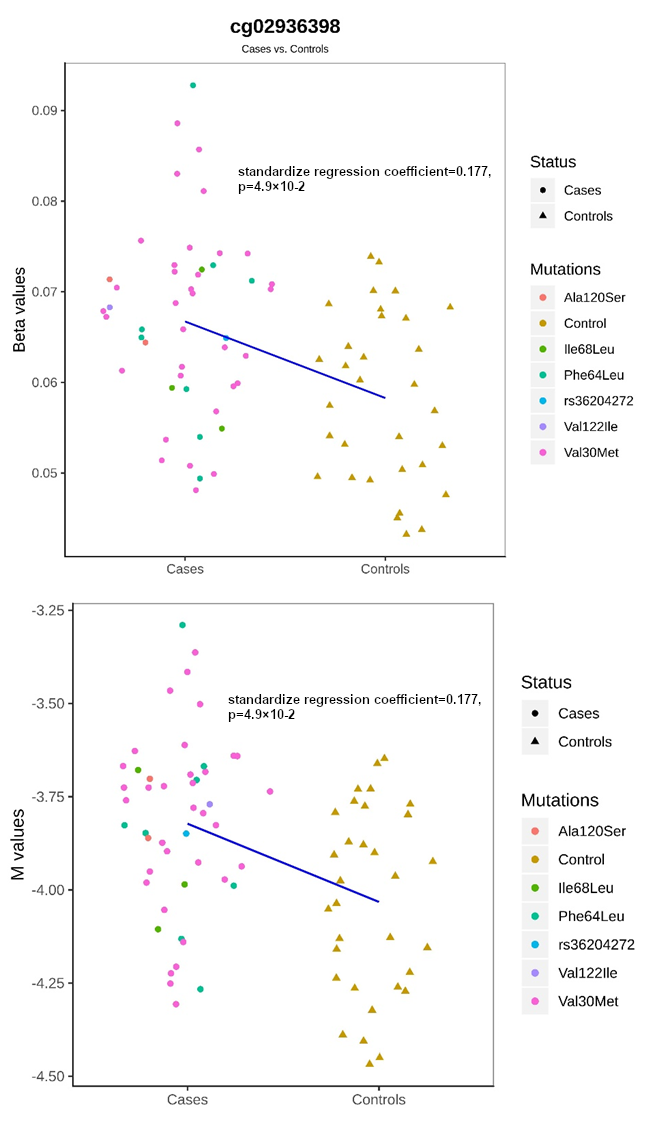


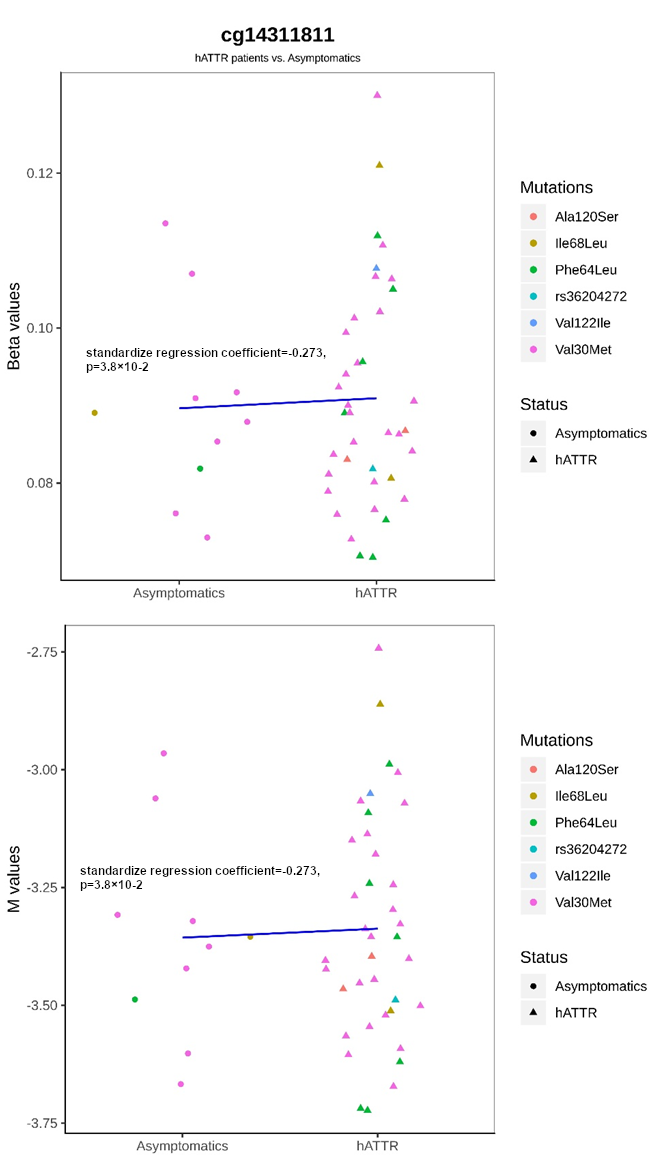


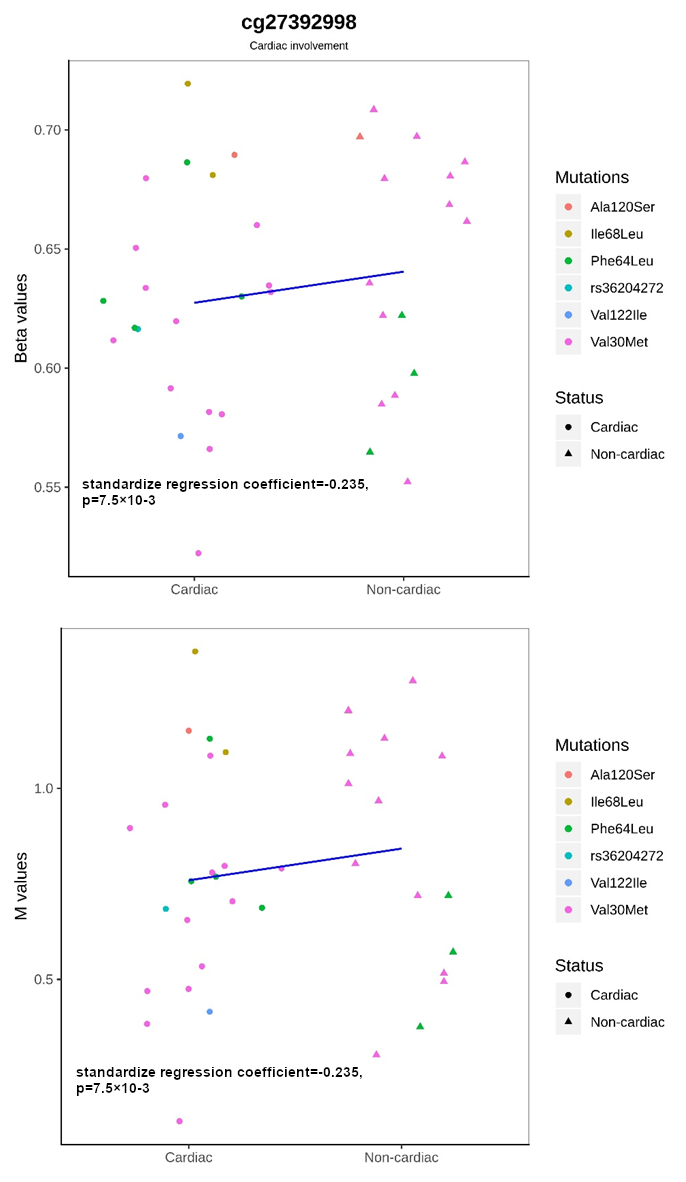


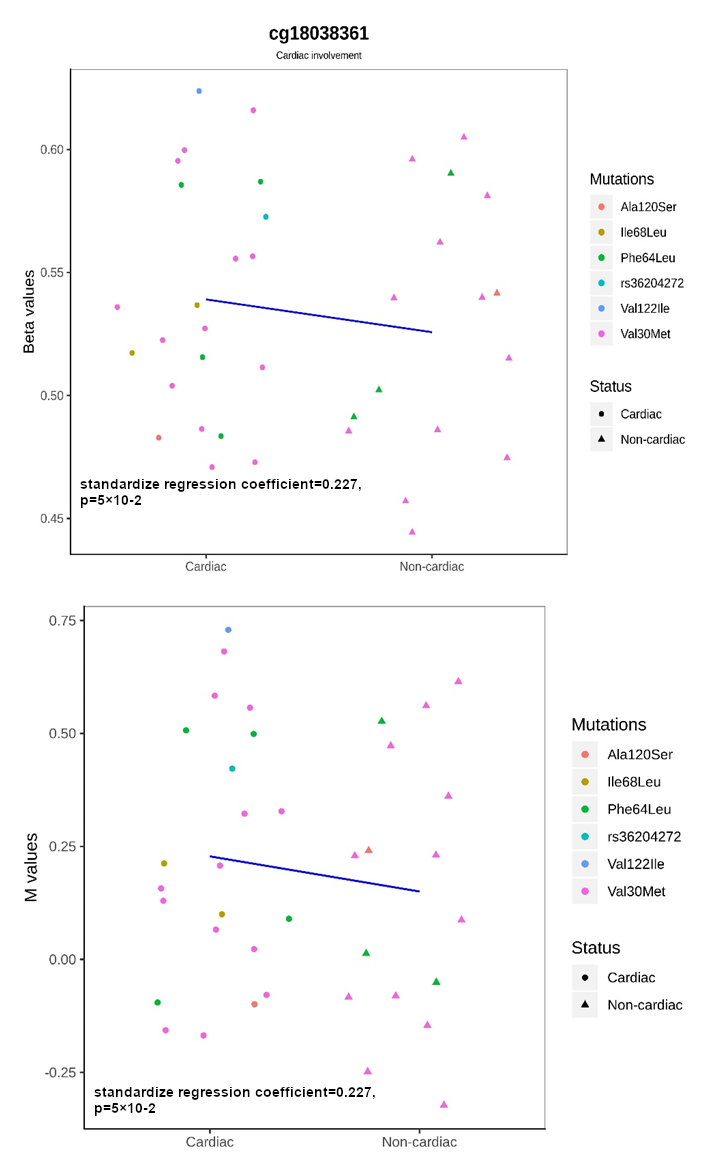


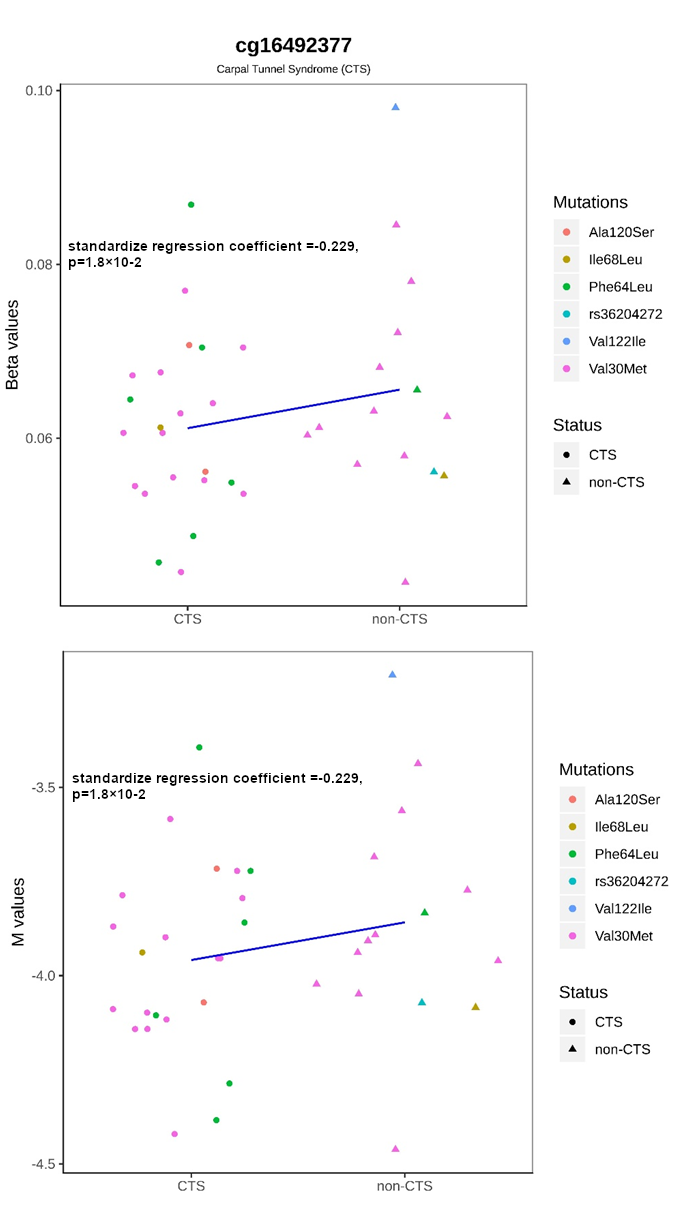


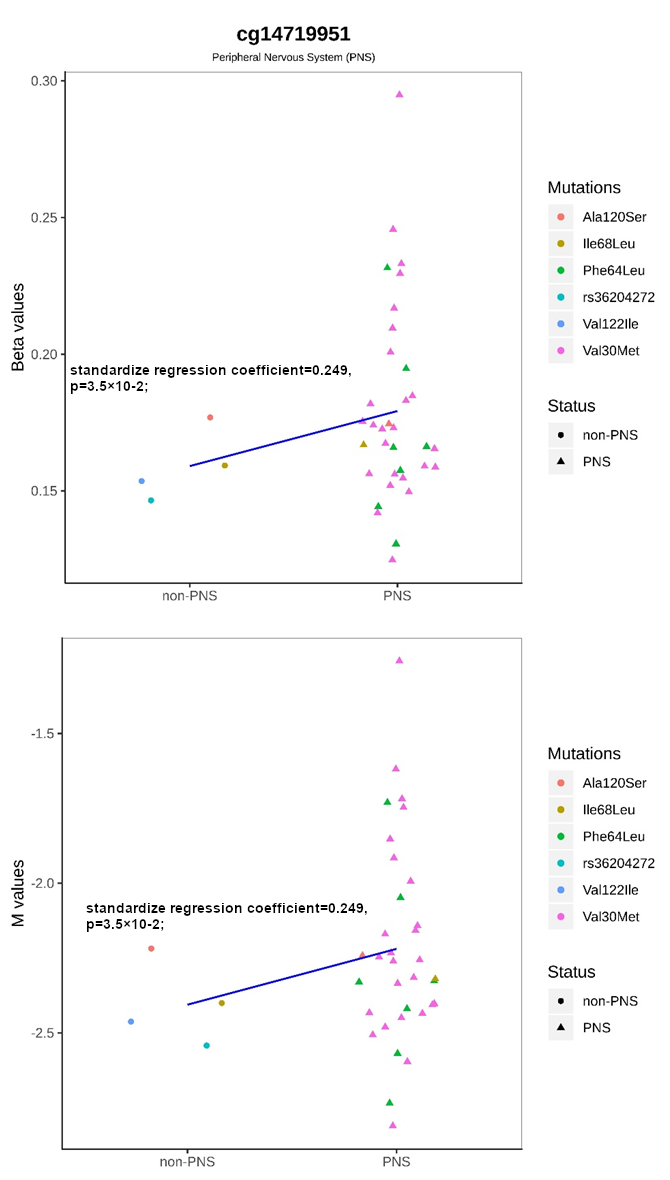

Supplement: Supplementary file 6 — Additional file 6. Methylation changes of co-methylated cg13139646-correlated CpG sites with respect to hATTR-related phenotypes: cg19203115, hATTR patients vs. asymptomatic carriers; cg11481443, cases vs. controls; cg02936398, cases vs. controls; cg14311811, hATTR patients vs. asymptomatic carriers; cg27392998, cardiac involvement in hATTR patients; cg18038361, cardiac involvement in hATTR patients; cg16492377, carpal tunnel syndrome in hATTR patients; cg14719951, peripheral nervous system involvement in hATTR patients. [file 13148_2020_967_MOESM6_ESM.docx]
