# Supplementary material for: Epigenetic profiling of Italian patients identified methylation sites associated with hereditary transthyretin amyloidosis
Source: Clin Epigenetics. 2020 Nov 17;12:176. doi: 10.1186/s13148-020-00967-6 (PMC7672937; doi:10.1186/s13148-020-00967-6)

**Additional File 7:** Statistical power calculations based on medium and small effect sizes (Δ_β_ = 0.5 and 0.2, respectively) and multiple sample sizes.


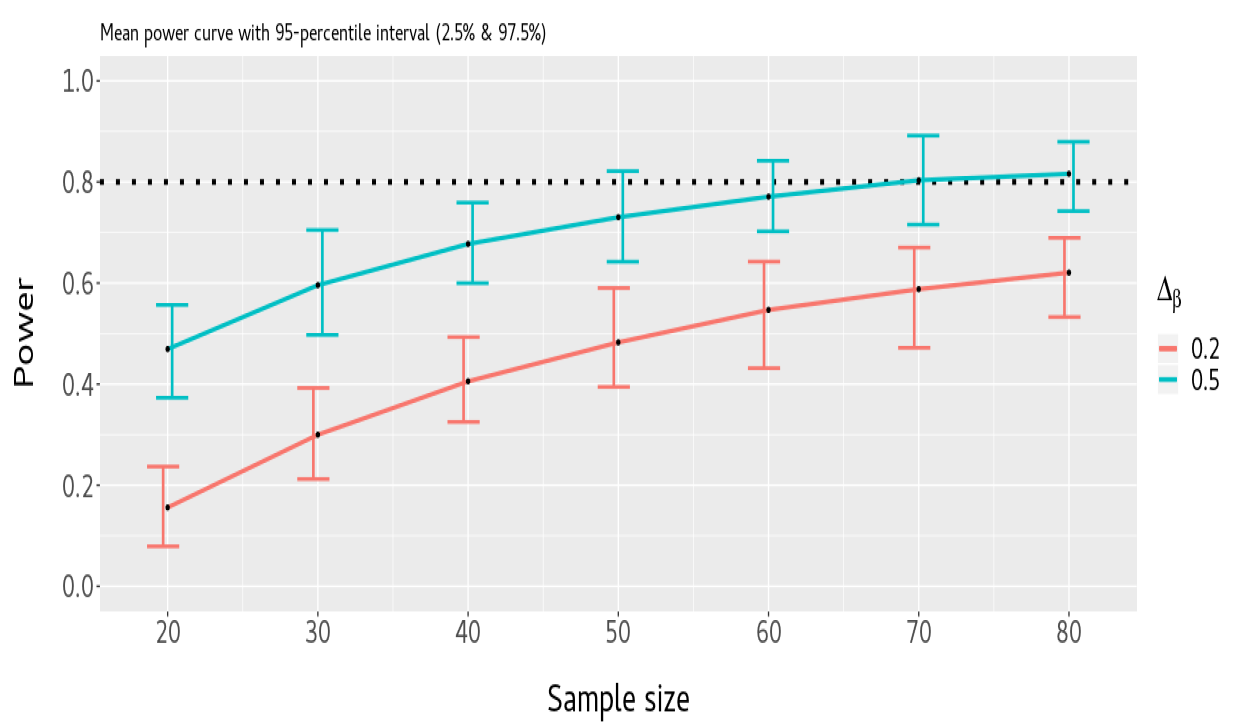

Supplement: Supplementary file 7 — Additional file 7. Statistical power calculations based on medium and small effect sizes (Δβ = 0.5 and 0.2, respectively) and multiple sample sizes. [file 13148_2020_967_MOESM7_ESM.docx]
